# Biochemical and Clinical Characterization of a Patient with Partial Biotinidase Deficiency Carrying the Rare BTD c.690C>G (p.Phe230Leu) Variant: A Case Report

**DOI:** 10.3390/life16091508

**Published:** 2026-09-10

**Authors:** Daniela Semeraro, Sara Verrocchio, Rossella Ferrante, Claudia Rossi, Damiana Pieragostino, Alex Zappacosta, Carlotta Buccolini, Silvia Di Michele, Luca Natale, Gessica Di Carlo, Cesidio Giuliani, Ines Bucci, Valentina Gatta

**Affiliations:** 1 Center for Advanced Studies and Technology (CAST), “G. d’Annunzio” University of Chieti-Pescara, 66100 Chieti, Italy; daniela.semeraro@unich.it (D.S.); sara.verrocchio@unich.it (S.V.); rossella.ferrante@unich.it (R.F.); claudia.rossi@unich.it (C.R.); damiana.pieragostino@unich.it (D.P.); luca.natale@unich.it (L.N.); gessica.dicarlo@phd.unich.it (G.D.C.); cesidio.giuliani@unich.it (C.G.); valentina.gatta@unich.it (V.G.); 2 Department of Medicine and Aging Science, “G. d’Annunzio” University of Chieti-Pescara, 66100 Chieti, Italy; 3 Department of Science, “G. d’Annunzio” University of Chieti-Pescara, 66100 Chieti, Italy; 4 Department of Innovative Technologies in Medicine and Dentistry, “G. d’Annunzio” University of Chieti-Pescara, 66100 Chieti, Italy; 5 Unit of Molecular Genetics, Center for Advanced Studies and Technology (CAST), “G. d’Annunzio” University of Chieti-Pescara, 66100 Chieti, Italy; alex.zappacosta@unidav.it (A.Z.); carlotta.buccolini@studenti.unich.it (C.B.); 6 Department of Humanities, Law and Economics, “Leonardo da Vinci” Telematic University, 66010 Torrevecchia Teatina, Italy; 7 Department of Neurosciences, Imaging and Clinical Sciences, “G. d’Annunzio” University of Chieti-Pescara, 66100 Chieti, Italy; 8 Department of Pediatrics, “Spirito Santo” Hospital, 65124 Pescara, Italy; silvia.dimichele@asl.pe.it

**Keywords:** biotinidase deficiency, expanded newborn screening, biotinidase gene variants, biotinidase activity

## Abstract

Biotinidase deficiency (BD) is an autosomal recessive inherited disorder in which the enzyme biotinidase (BTD) is totally or partially defective and the vitamin biotin is not recycled. More than 200 pathogenic variants in the BTD gene have been identified, causing complete or partial loss of enzyme activity and leading to profound or partial BD. Although the genotype–phenotype correlation is not fully defined, some genotypes are associated with profound forms and others with partial forms of BD. The patients with partial BD are mostly asymptomatic but symptoms may appear during stressful conditions such as infections. The patients with profound BD may present neurological problems, hearing loss, visual abnormalities, and organic aciduria, if not treated with biotin supplementation. BD is included in most newborn screening programs (NBS). Screening test relies on measurement of BTD enzyme activity in dried blood spot (DBS), followed by a plasma BTD assay and BTD molecular analysis as confirmatory test. We describe a male newborn with partial BD identified through the newborn screening program. Low BTD activity on DBS was detected; BTD molecular analysis showed the proband to be compound heterozygous for the c.1270G>C (p.Asp424His) mild known variant, and for the rare variant c.690C>G (p.Phe230Leu) previously identified by our group. In this study, we provide the biochemical and clinical characterization of a patient carrying the c.690C>G (p.Phe230Leu) variant, and our findings support a possible contribution of this variant to the partial BD phenotype.

## 1. Introduction

**Figure 1:**
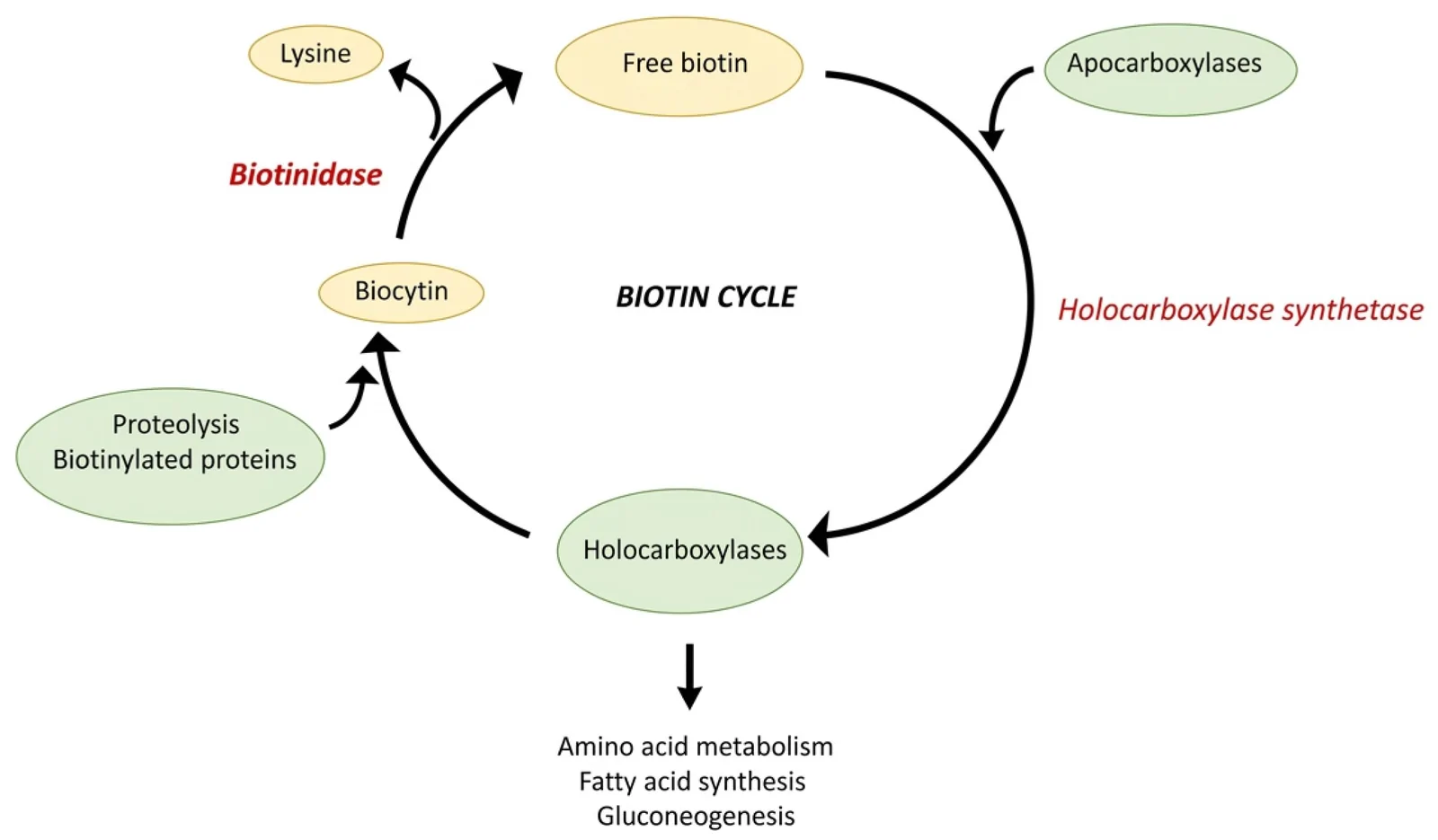
**The biotin** cycle: free biotin is covalently bound by holocarboxylase synthetase to the active sites of apocarboxylase: pyruvate carboxylase (PC), acetyl-CoA carboxylase (ACC), propionyl-CoA carboxylase (PCC) and 3-methylcrotonyl-CoA carboxylase (MCC). These carboxylases, which are essential for amino acid metabolism, fatty acid synthesis and gluconeogenesis, are degraded into biocytin or small biotinylated peptides after fulfilling their biological roles. **Biotinidase** subsequently cleaves these compounds into **free biotin** and **lysine**, thereby recycling the vitamin.

Biotinidase deficiency (BD; OMIM 253260), also known as late-onset multiple carboxylase deficiency, is an autosomal recessive metabolic disorder caused by pathogenic variants in the BTD gene, which encodes the biotinidase enzyme (E.C. 3.5.1.12). This enzyme is essential for recycling biotin, a water-soluble B-complex vitamin that acts as a cofactor for four carboxylases: pyruvate carboxylase, propionyl-CoA carboxylase, methylcrotonyl-CoA carboxylase, and acetyl-CoA carboxylase. These enzymes are involved in gluconeogenesis, amino acid catabolism, and fatty acid synthesis. Biotinidase releases free biotin from biocytin and biotinylated peptides generated during proteolytic degradation, maintaining intracellular biotin levels (Figure 1) [1].

BD is classified into two forms based on residual enzyme activity: profound (<10% of mean normal activity) and partial (10–30%) [2,3].

The BTD gene, located on chromosome 3p25, is ubiquitously expressed, and more than 200 pathogenic variants have been identified. A common pathogenic mild allele, NM_001370658.1(BTD):c.1270G>C (p.Asp424His), historically reported as NM_000060.4(BTD):c.1330G>C (p.Asp444His), reduces enzyme activity by approximately 50% in homozygous individuals. In compound heterozygotes with a profound variant, it results in partial BD, with residual activity around 20–25%. Over 98% of individuals with partial BD carry this variant on one allele [4].

The global incidence of BD ranges from 1:40,000 to 1:60,000 live births [5] with higher rates reported in Brazil (1:9000) [6] and in countries with high consanguinity such as Turkey and Saudi Arabia [3,7,8,9,10]. In Italy, incidence shows regional variability, ranging from approximately 1:35,000 [11] to 1:6000 [12,13,14]. Clinically, untreated profound BD typically presents between 2 and 5 months of age with neurological and dermatological symptoms, including seizures, hypotonia, skin rash, and alopecia. Additional manifestations include ataxia, respiratory abnormalities, developmental delay, conjunctivitis, hearing loss, and optic atrophy [4,15,16]. Severe cases may develop metabolic ketoacidosis and organic aciduria, potentially leading to coma or death. Partial BD is generally milder and may become symptomatic only during metabolic stress, such as infection or fasting [17,18]. Consequently, BD meets criteria for inclusion in newborn screening (NBS) programs. Screening is performed by measuring BTD enzyme activity in dried blood spots (DBS), with confirmatory diagnosis based on serum or plasma enzyme activity and molecular genetic analysis of the BTD gene. In Italy, expanded newborn screening is mandatory and is offered free of charge to all newborns, BD is included among the disorders covered by the national expanded newborn screening program. In a previous study, we reported a high incidence of partial BD in our regional NBS program and identified the rare BTD variant c.690C>G (p.Phe230Leu) [14]. However, the biochemical and clinical significance of this variant remained unclear. In the present study, we provide a detailed biochemical and clinical characterization of a patient carrying c.690C>G (p.Phe230Leu), with the aim of further investigating its potential contribution to the partial BD phenotype.

## 2. Case Presentation

### 2.1. Case Description

The patient, a male newborn was born at 39 weeks of gestation age by Caucasian non-consanguineous parents, the birth weight was adequate for the gestational age (3760 g). Neonatal parameters were unremarkable.

BD newborn screening was performed at the Center for Advanced Studies and Technology (CAST) of the University of Chieti-Pescara, as a part of the Italian NBS core panel [14]. According to the laboratory protocol, screening test for BD relies on measuring BTD activity in DBS samples by a semi-quantitative time-resolved immunofluorescence method on a fully automated integrated screening plate processor (GSP^®^ Neonatal Biotinidase kit, Revvity, Wallac Oy, Turku, Finland).

The cut-off of the BTD enzyme activity is set at 85 U/dL (0.5th percentile of the population distribution). For any result below 85 U/dL, a recall sample DBS is requested. If the BTD activity on the recall sample is still below the cut-off, the patient is suspected of BD and referred to the Pediatric Department of the Santo Spirito Hospital in Pescara for clinical evaluation and confirmatory tests.

The newborn tested positive at the NBS performed on the second day of life due to reduced BTD activity (62.1 U/dL). The newborn was recalled. Following recall, the repeat test confirmed low enzymatic activity (59.2 U/dL), and the newborn was subsequently referred to the medical team for further evaluation.

The patient underwent biochemical and clinical workup: blood count with formula, biochemical profile with lactate, ammonia, blood gas analysis, audiometry, eye examination, growth, and neurological development evaluation as well as skin examination. At the initial clinical evaluation, no signs or symptoms related to BD were observed. Psychomotor development and auditory and visual functions were normal, and no relevant dermatological problems attributable to BD were detected. An additional DBS was collected, and the BTD activity was still low (57,6 U/dL). The mean BTD activity in the infant was 59.5 U/dL, corresponding to approximately 25% of the mean activity established from a reference population of at least 2500 newborns with normal screening results and periodically reassessed on an annual basis using screening data (232 U/dL).

### 2.2. Molecular Analysis

Genomic DNA was extracted from peripheral blood of the proband and his parents, using MagPurix Blood DNA Extraction Kit 200 (Resnova, Rome, Italy), following the manufacturer’s protocol.

Polymerase chain reaction (PCR) was performed, and sequencing reaction was performed using BigDye Terminator™ v1.1 Cycle Sequencing Kit (Applied Biosystems by Thermo Fisher Scientific, Schwerte, Germany). Sequencing was carried out using SeqStudio Genetic Analyzer (Applied Biosystems by Thermo Fisher Scientific, Schwerte, Germany).

Sequence data were analyzed using the BTD reference transcript NM_001370658.1 available in the National Center for Biotechnology Information (NCBI) GenBank database. Control samples from unrelated unaffected individuals of the same ethnic background were obtained from a previously established sample collection. These samples had been collected in accordance with institutional procedures and applicable ethical requirements, and written informed consent covered the use of biological samples and genetic data for subsequent research analyses. The c.690C>G (p.Phe230Leu) variant was not detected in the control samples analyzed. No additional samples were collected specifically for the purposes of the present case report.

Sanger sequencing of the BTD gene identified two variants: NM_001370658.1(BTD):c.1270G>C (p.Asp424His), a well-established mild BTD variant, and NM_001370658.1(BTD):c.690C>G (p.Phe230Leu). The c.690C>G (p.Phe230Leu) variant (rs2125501783) is extremely rare in population databases (gnomAD allele frequency: 0.00001239) and is currently classified in ClinVar as a variant of uncertain significance (VUS; ClinVar accession VCV003251568.1; one-star review status, criteria provided, single submitter, Women’s Health and Genetics/Laboratory Corporation of America [Labcorp]) [19,20]. Segregation analysis in the parents demonstrated that c.690C>G (p.Phe230Leu) was paternally inherited, whereas c.1270G>C (p.Asp424His) was maternally inherited, confirming that the two variants are in trans. According to the ACMG/AMP criteria, c.690C>G (p.Phe230Leu) was classified as a variant of uncertain significance (VUS).

According to the ACMG/AMP criteria, c.690C>G (p.Phe230Leu) was classified as a variant of uncertain significance (VUS). Its extremely low frequency in population databases supports the application of PM2_Supporting. Segregation analysis confirmed that c.690C>G (p.Phe230Leu) occurs in trans with c.1270G>C (p.Asp424His). The latter is a well-characterized mild BTD allele associated with partial BD when present in trans with a severe pathogenic BTD variant. Given the specific genotype–phenotype relationship of c.1270G>C and its limited phenotypic effect in the homozygous state, PM3 was conservatively applied at Supporting strength. In the absence of direct variant-specific functional studies or additional unrelated affected individuals carrying c.690C>G, the currently available evidence remains insufficient to support reclassification as likely pathogenic. The proband is therefore a compound heterozygote for the two BTD variants, consistent with the observed biochemical phenotype.

**Figure 2:**
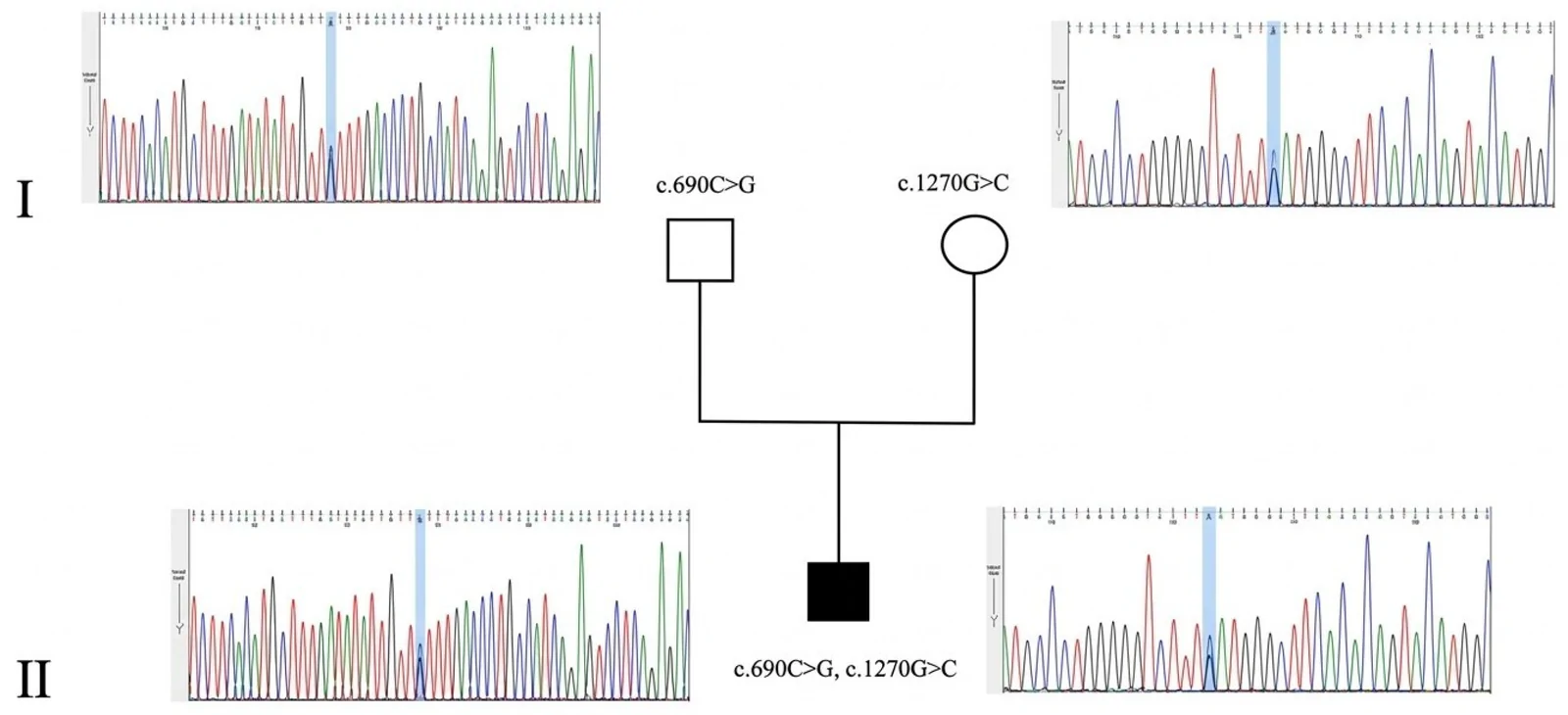
Family pedigree (**center**) showing the segregation of the two identified BTD gene variants across generations (I: parents; II: proband). Representative Sanger sequencing electropherograms illustrate the heterozygous c.690C>G variant of paternal origin (**top left**) and the heterozygous c.1270G>C variant of maternal origin (**top right**). The proband (affected male, filled square) inherited both variants in trans (c.690C>G and c.1270G>C; **bottom** electropherograms), confirming compound heterozygosity.

This configuration is clinically relevant in autosomal recessive disorders such as BD, where pathogenic variants must affect both alleles to significantly impair enzyme activity. Consequently, the proband is a compound heterozygote, a carrier of two different variants on separate parental chromosomes (in trans), a genetic configuration consistent with the observed biochemical phenotype (Figure 2).

### 2.3. Plasma BTD Activity

A blood sample was collected from the patient at 18 months of age at the clinical reference center. Plasma BTD activity measurement was not routinely performed at the beginning of the newborn screening program, when diagnosis was based on DBS BTD activity and molecular genetic analysis. Once introduced into our protocol, plasma BTD activity was also assessed during follow-up in previously diagnosed patients. Specimen collection, transport, storage were performed according to current guidelines [5], and the plasma was analyzed upon arrival at the laboratory. BTD activity was measured by a colorimetric assay using the N(+)-biotinyl-4-aminobenzoic acid (B-PABA) as the substrate (Biotinidase serum/plasma, LTA Milan, Italy). According to the manufacturer, the values are classified as follows: normal range 4.4–12 nmol/min/mL; partial BTD deficiency 0.7–2.1 nmol/min/mL; and heterozygous range 2.2–5.2 nmol/min/mL. The assay does not distinguish between age groups, samples from positive and normal individuals were contextually analyzed [8]. The BTD activities measured in the normal and positive controls included in the assay were 7.5 and 1.8 nmol/min/mL, respectively. The sample was analyzed in duplicate.

The plasma BTD activity of the infant was 1.9 nmol/min/mL, in the range of partial BTD deficiency. The results were consistent with the corresponding neonatal DBS findings. These biochemical findings are consistent with a possible functional impact of the c.690C>G (p.Phe230Leu) variant in the compound heterozygous state.

### 2.4. Clinical Course and Follow-Up

Following diagnostic confirmation, the patient entered regular clinical follow-up. Initially, biotin therapy was not initiated, except during periods of stress or fever, during which biotin treatment (5 mg/day) was recommended. This approach reflected our clinical practice at that time, in the context of the variability in management strategies for partial BD reported in the literature [3]. Our management strategy subsequently evolved toward continuous biotin supplementation in patients with partial BD, in line with recommendations favoring treatment in affected individuals [2]. During follow-up, the child demonstrated normal motor development, achieving independent walking at 12 months. At 18 months of age, concerns regarding attention and mild slowing in language development were reported by the parents and noted during routine clinical follow-up. Although no standardized developmental assessment tools were administered, these observations supported the decision to initiate continuous biotin supplementation (5 mg/day) at 18 months of age, with the aim of minimizing any potentially contributing metabolic factor; however, a causal relationship between partial BD and the developmental findings could not be established. Speech therapy was initiated concurrently, and an improvement was observed during subsequent follow-up. The patient is currently 6 years and 4 months old. At the most recent follow-up visit, performed at 6 years of age, the patient exhibited normal growth (25th percentile for weight and 25th–50th percentile for height). Ophthalmologic examination revealed no pathological findings, and audiological assessment was within normal limits. No cutaneous manifestations have ever been observed.

## 3. Discussion

We report a proband who was found to be compound heterozygous for the NM_001370658.1(BTD) c.1270G>C, a well-established mild BTD variant [21], and c.690C>G variant (p.Phe230Leu). This variant that we previously described, reported in ClinVar Database as a variant of uncertain significance, results in a missense substitution, p.Phe230Leu, affecting a highly conserved residue of the biotinidase protein. The c.690C>G variant is extremely rare in population databases (gnomAD allele frequency: 0.00001239) and is currently classified as a variant of uncertain significance in ClinVar. However, several lines of evidence support a possible functional contribution of this variant to the partial BD phenotype.

Partial BD usually occurs when an individual has one allele that results in nearly total loss of activity in combination with an allele having the c.1270G>C variant and is characterized by approximately 10–30% of normal enzyme activity [21].

The approximately 25% residual BTD activity observed in the proband is consistent with the biochemical phenotype typically reported in individuals carrying c.1270G>C (p.Asp424His) in trans with a deleterious BTD variant. This genotype–phenotype relationship has been consistently reported in the literature and was also observed in our previously published newborn screening cohort. Although this finding supports a possible functional impact of c.690C>G (p.Phe230Leu), it does not allow the functional effect of this variant to be quantified independently [8].

Segregation analysis showed that both variants are in trans, which means the proband is a true compound heterozygote. Individuals who are compound heterozygous for one mild and one severe BTD variant usually present with partial BD and are frequently identified through newborn screening and subsequently treated with oral biotin supplementation [22]. A number of investigators have suggested reconsidering the assessment of BTD enzymatic activity at 12 months, at 1 year, or following 5 years [23,24].

In conclusion we demonstrated partial BTD activity in both DBS and plasma in a patient carrying c.690C>G (p.Phe230Leu) in trans with the well-established mild c.1270G>C (p.Asp424His) variant, supporting a possible contribution of c.690C>G to the partial BTD deficiency phenotype. However, data from additional unrelated patients carrying the same variant, as well as direct functional studies, are required to conclusively establish its pathogenic role.

New gene variants continue to be identified and characterized, underscoring the importance of periodically reassessing the genotype–phenotype relationship and residual enzymatic activity throughout the years. This expansion underscores the significance of re-evaluating genotype–phenotype correlations and monitoring residual enzyme activity over the course of time, since changes in measured enzyme activity may be observed with age, potentially leading to a change in biochemical classification.

Follow-up evaluation is particularly important in subjects with mild heterozygous or compound genotypes, in whom enzymatic activity may vary between partial deficiency, profound deficiency, and carrier status. Continuing clinical and biochemical follow-up, together with current interpretation of the variants is essential to improve disease classifications and treatment options.

## Data Availability

Due to privacy restrictions, the data presented in this study are only available on request from the corresponding author.

## Abbreviations

The following abbreviations are used in this manuscript:

BD	Biotinidase Deficiency
BTD	Enzyme Biotinidase
NBS	Newborn Screening Programs
DBS	Dried Blood Spot
CAST	Center for Advanced Studies and Technology
PCR	Polymerase Chain Reaction
PC	Pyruvate Carboxylase
ACC	Acetyl-CoA Carboxylase
PCC	Propionyl-CoA Carboxylase
MCC	Methylcrotonil-CoA Carboxylase
NCBI	National Center for Biotechnology Information
